# Investigating Annual Diving Behaviour by Hooded Seals (*Cystophora cristata*) within the Northwest Atlantic Ocean

**DOI:** 10.1371/journal.pone.0080438

**Published:** 2013-11-25

**Authors:** Julie M. Andersen, Mette Skern-Mauritzen, Lars Boehme, Yolanda F. Wiersma, Aqqalu Rosing-Asvid, Mike O. Hammill, Garry B. Stenson

**Affiliations:** 1 Department of Biology, Memorial University of Newfoundland, St. John’s, Newfoundland and Labrador, Canada; 2 Marine Mammal Research Department, Institute of Marine Research, Bergen, Norway; 3 Sea Mammal Research Unit, Scottish Oceans Institute, University of St. Andrews, St. Andrews, United Kingdom; 4 Greenland Institute of Natural Resources, Nuuk, Greenland; 5 Science Branch, Department of Fisheries and Oceans, Institute du Maurice Lamontang, Mont Joli, Quebec, Canada; 6 Science Branch, Department of Fisheries and Oceans, Northwest Atlantic Fisheries Centre, St. John’s, Newfoundland and Labrador, Canada; University of Pretoria, South Africa

## Abstract

With the exception of relatively brief periods when they reproduce and moult, hooded seals, *Cystophora cristata,* spend most of the year in the open ocean where they undergo feeding migrations to either recover or prepare for the next fasting period. Valuable insights into habitat use and diving behaviour during these periods have been obtained by attaching Satellite Relay Data Loggers (SRDLs) to 51 Northwest (NW) Atlantic hooded seals (33 females and 18 males) during ice-bound fasting periods (2004−2008). Using General Additive Models (GAMs) we describe habitat use in terms of First Passage Time (FPT) and analyse how bathymetry, seasonality and FPT influence the hooded seals’ diving behaviour described by maximum dive depth, dive duration and surface duration. Adult NW Atlantic hooded seals exhibit a change in diving activity in areas where they spend >20 h by increasing maximum dive depth, dive duration and surface duration, indicating a restricted search behaviour. We found that male and female hooded seals are spatially segregated and that diving behaviour varies between sexes in relation to habitat properties and seasonality. Migration periods are described by increased dive duration for both sexes with a peak in May, October and January. Males demonstrated an increase in dive depth and dive duration towards May (post-breeding/pre-moult) and August–October (post-moult/pre-breeding) but did not show any pronounced increase in surface duration. Females dived deepest and had the highest surface duration between December and January (post-moult/pre-breeding). Our results suggest that the smaller females may have a greater need to recover from dives than that of the larger males. Horizontal segregation could have evolved as a result of a resource partitioning strategy to avoid sexual competition or that the energy requirements of males and females are different due to different energy expenditure during fasting periods.

## Introduction

**Figure 1:**
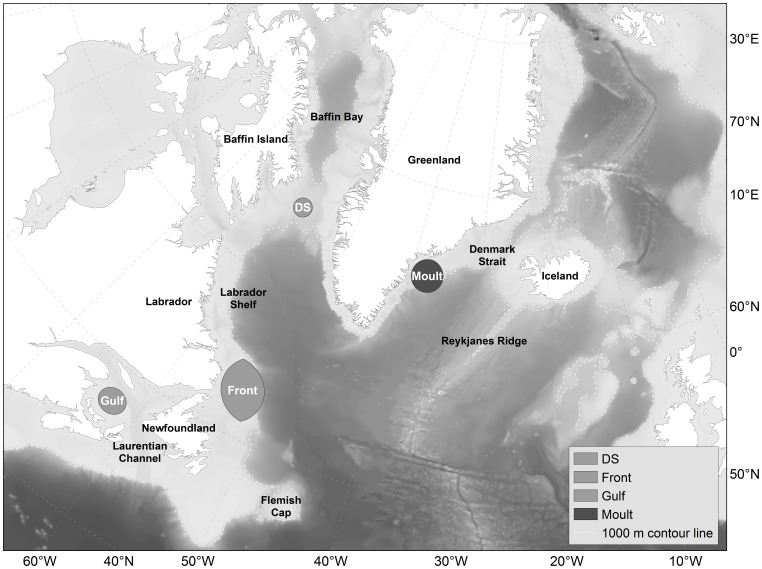
Map over the study area. Moulting area is located in southeast Greenland and breeding areas in Davis Strait (DS), off Labrador and Newfoundland (Front) and in the Gulf og St. Lawrence (Gulf). Bathymetry of the study area is presented as backdrop in grey scale. Dashed white line is the 1,000 meter contour.

The Northwest (NW) Atlantic Ocean is a highly dynamic and productive oceanographic system that is influenced by a number of currents (the East Greenland Current, West Greenland Current and the Labrador Current) in conjunction with cross shelf exchange between warmer continental slope water and colder water via sea bottom topography [1]–[2]. The NW Atlantic hooded seal (*Cystophora cristata*) is distributed throughout these waters displaying a distinct annual migration pattern [3]–[4]. They leave their whelping grounds in the Davis Strait, off Newfoundland and Labrador (the Front) and the Gulf of St. Lawrence (the Gulf) by late March, and disperse along slope edges to feed ([3], [5], Fig. 1). Toward the end of their post-breeding/pre-moult feeding period they arrive at the ice off southeast (SE) Greenland where they moult during July [3]–[6]. After the moult, which is highly synchronous, they disperse northwards along the west Greenland shelf and across the Labrador Sea and Baffin Bay (post-moult/pre-breeding period) before returning to their respective whelping areas [3]–[4].

Like most phocids, hooded seals are capital breeders. They fast during pupping and breeding, relying on energy reserves obtained during the post-moult/pre-breeding period [7]. The moult is also a potentially energetically expensive fasting period for phocids (e.g., harbour seals (*Phoca vitulina*), [8], grey seals (*Halichoerus grypus*), [9], southern elephant seals (*Mirounga leonina*), [10]–[11] ) and the extent of body mass loss in hooded seals during this period has been estimated to be as high as 14% [12].

Hooded seals are sexually dimorphic animals with males weighing, on average, 250 kg and females 190 kg, although they can get much larger (up to 460 kg for males and 300 kg for females) [13]–[15]. Sexually dimorphic animals have been found to display differences in spatial distribution and diet preferences (e.g., [5], ). Males must acquire more resources to attain, and maintain, their greater size [21]–[22], especially when preparing for the breeding season and competition for females. Males have been found to lose 14% of their mean body mass (∼2.5 kg per day) over a breeding period lasting 2.5 weeks [24]. In comparison, females need to attain energy stores to maintain pregnancy and prepare for a very short, but intense, lactation period. Female hooded seals wean their pup in 3−5 days during which time they lose 16% of their mean body mass (∼10 kg per day) [25]–[26]. Thus, the total body mass of these animals fluctuates throughout the year in relation to important life history events.

Optimal foraging theory predicts that organisms should spend more time in areas where resources are plentiful than in areas where resources are scarce [27]–[28]. The same theory predicts that breath-hold divers should adjust time allocation within their dives to the distance separating prey from the surface [29]. As hooded seals carry out their extensive migrations each year, the specific movement patterns and diving behaviour should, to some extent, reflect the distribution and availability of their prey [30]–[31]. Diet studies have shown that hooded seals mainly forage on benthopelagic prey consisting of species such as Atlantic halibut (*Hippoglossus hippoglossus*), Greenland halibut (*Reinhardtius hippoglossoides*), redfish (*Sebastes sp*.), squid (*Gonatus fabricii*), herring (*Clupea harengus*), capelin (*Mallotus villosus*), Atlantic cod (*Gadus morhua*), Arctic cod (*Boreogadus saida*), blue hake (*Antimora rostrata*), and white baraccudine (*Arctozenus rissoi,* [13], . Furthermore, diet variation between sex, age and season has been observed [13], [35]. Recent research on the highly sexually dimorphic southern elephant seal suggests that differences in dive depth appear to be caused by differences in prey selection between sexes [36]. This indicates that southern elephant seals are using a resource partitioning strategy and that they may be displaying avoidance behaviour as a result of inter-sexual competition [36]. Previous research on NW Atlantic hooded seals has found that males and females are spatially segregated [4]–[5], which could indicate that hooded seals also use a similar foraging strategy to avoid competition.

Satellite telemetry has proven to be an especially useful tool in monitoring pelagic marine mammals (e.g., [5], [30], ). The availability of data on the movements of free-ranging marine mammals has led to the adoption of powerful analytic approaches for the investigation of habitat use, which in turn allows for researchers to gain insight into possible foraging strategies (e.g., [27], [37], ). One method to analytically investigate habitat selection and use is to incorporate first passage time (FPT) as part of habitat modelling (e.g., [27], [37], [45], [48]). FPT is defined as the time it takes an animal to cross a circle of a given size, which is based on the animal’s average area-restricted search (ARS) scale [27]. ARS occurs when an animal responds to an abundant food source by slowing down and increasing its turning rate [50]–[51]. This simple behavioural response increases the likelihood that the animal can better exploit a patch of food, as prey encounters will be more frequent [51]. The animal will not continue on a wider ranging search until prey encounter rate decreases [51]. FPT can therefore be used as a scale-dependent measure of habitat selection and as an indication of feeding effort, since search effort can be expected to be higher in habitats of high prey encounter rate.

Habitat use by NW Atlantic hooded seals has previously been investigated in a two dimensional landscape. Andersen et al. [4] identified FPT along the migration track and related location data at the surface to environmental parameters such as bottom depth, slope, sea surface temperature, surface chlorophyll and ice concentrations. However, marine environments are characterized by three dimensions, which are fully exploited by marine mammals. Analysing data on diving behaviour in areas of various FPTs can offer valuable indirect information on hooded seal habitat selection and foraging strategies in the NW Atlantic Ocean.

Andersen et al. [4] showed that hooded seals had longer FPT in SE Greenland (moulting area), the Gulf (high use area for animals breeding there) and in the Davis Strait area. Males had longer FPT in Baffin Bay, while females had longer FPT along the Labrador shelf and Reykjanes Ridge area. Therefore, even though there was some spatial overlap between sexes, spatial and temporal differences in habitat use and geographic locations were observed [3]–[4]. Among females, long FPTs were associated with intermediate bottom depths along the shelf break and areas with high primary productivity. Males appeared to be more spatially restricted than females and were associated with complex seafloor relief and cool surface temperatures [4].

Here we will build on previous research to describe the diving behaviour of these same seals in relation to habitat. As a sexually dimorphic species we will investigate (i) if males and females exhibit differences in seasonal diving behaviour and FPT in relation to spatial location and bathymetry and (ii) if these characteristics vary in relation to the annual breeding and moulting periods. This knowledge can improve our understanding of the overall habitat use and foraging strategies of hooded seals in the NW Atlantic Ocean, and thereby offer valuable information that can improve future management of the species.

## Methods

### Ethics statement

The capture and tagging protocols have been reviewed and approved by the Canadian Council of Animal Care (Newfoundland Region). Capture and deployment of satellite transmitters was carried out under annual animal care permits and by experienced personnel with the Department of Fisheries and Oceans (DFO), Canada. The CCAC permit numbers are NAFC 2004-11 and NAFC 2008-04.

### Study area and deployment of Satellite Relay Data Loggers (SRDLs)

The study area extends throughout the NW Atlantic Ocean from the Gulf of St. Lawrence and the Flemish cap in the south to the Denmark Strait in the east and Baffin Bay to the north (Fig.1).

Satellite transmitters were deployed directly after the annual moult in SE Greenland during three field seasons (2004, 2005 and 2007, 65°N, 37°W), and after the whelping and breeding period during three field seasons (The Front: 2004 and 2008, 49°N, 52°W, The Gulf: 2004, 2005 and 2008, 46°50′N, 62°W). In total, data from 51 tagged seals were used in this study (33 adult females and 18 adult males). The animals were captured using a net. They were then weighed, and subsequently tranquillised using an intramuscular administration of tiletamine hydrochloride and zolazepam hydrochloride (1 mg. 100 kg^−1^; Telazol, AH. Robins Company, Richmond, VZ, USA). Satellite Relay Data Loggers (SRDLs, Sea Mammal Research Unit (SMRU), St. Andrews, Scotland) were glued to the head or upper neck by use of quick drying epoxy glue (Cure 5, Industrial Formulators of Canada Ltd. Burnaby, BC Canada).

The SRDLs collect a range of behavioural information about marine mammals at sea which is compressed and transmitted via the ARGOS satellite system [52]–[54]. The data collected included number of dives (dives deeper than 6 meters were recorded), dive depth, dive duration and surface intervals [52]–[53]. Transmissions were attempted every 80 seconds when the seals were at the surface.

### Habitat data

Habitat use was investigated by evaluating the association of individual annual movement patterns (based on diving location data) with seasonality (represented by month) and environmental variables, such as geographic location, bottom depth and the topographic complexity index (TCI).

Bottom depths at dive locations were extracted using the 1 minute bathymetry raster data from the General Bathymetry Chart of the Ocean (GEBCO, http://www.gebco.net/). TCI was calculated based on methods described by Wolock and McCabe [55] using GEBCO data, and an AML code in ArcInfo (version 9.3) by S. Wilds, modified by J. Young and F. van Manen, USGS LSC. The TCI model calculates (from each grid cell) the total upslope area, before calculating how much flow/drainage from the surrounding area that would accumulate in each grid cell, thereby offering a more realistic picture of the sea floor. The underlying formula is TCI  =  ln[(A/tanB], where A is the surface area of each grid cell providing “drainage” and B is the surface slope of the grid cell [55]–[56]. The TCI value of a grid cell is therefore dependent on the slope or shape of the sea floor in either direction (up or down) to the surrounding grid cells, which is represented by the terms flow or drainage. TCI identifies basins and peaks where high numeric values represent peaks, and low values represent basins (our data ranged from 0.53−25.32, where values around 12.5 represent a flat surface).

### Variation in First Passage Time (FPT)

We calculated the FPT radius by using the “adehabitatLT” package in R (version R 2.14.1, [57]). The spatial dynamics of foraging areas can be studied by analysing the spatial distribution of FPT among individuals [27]. To do this it is necessary to select a scale on which the FPT is to be calculated [48]. By creating a histogram of the variance of FPT vs. radius, we derived the size of the area in which the animals focus their search effort (ARS). The variance-scale function, and consequently the observed ARS scale, is related to individual foraging patterns and success as well as the spatial distribution of resources [58]. In order to remove some of the noise due to stochastic and individual differences in ARS scales we chose to use a common spatial scale (see [48] for details). Andersen et al. [4] estimated a search radius of 37.5 km for males, and 27.5 km and 37.5 km for females (post-breeding/pre-moult, post-moult/pre-breeding, respectively). Because high foraging success in some of the trips will mask a large-scale search pattern, we chose a spatial scale (40 km) in the upper range of individual ARS scales. The purpose of the FPT is to capture the search effort at each point along the entire movement path [27], [48]. With location data sampled independently of speed along a path, a larger number of location points will be sampled in areas of low speed compared to areas of high speed [27]. This gives a sampling bias toward search effort. In order to remove this bias, sampling points were made regular in space by spatial interpolation of locations [27]. Data points were placed 500 meters apart along each of the tracks and the data averaged per 80 km step (diameter of ARS circle) to obtain summary information about diving behaviour along the trajectory on the ARS scale.

FPT, extracted at the ARS scale, was used as a response variable in the GAM analysis (package “mgcv” in R, [59]), with geographic location, bottom depth, month and TCI as predictors (we refer to this as the “habitat model”). This was done to investigate how FPT could be explained by these habitat properties, and how FPT fluctuated annually. March and July were excluded from the analysis as hooded seals spend most of their time during these two months hauled out on the ice for breeding and moulting, respectively. To take into account individual variability, individual seal id was entered as a random factor using a smooth specifier [60]. The model is given by: 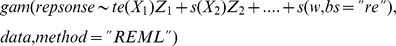



where FPT is the response variable and x_1_, x_2_ etc. are the predictor variables (geographic location, bottom depth, month and TCI). The models were run by including sex as an indicator variable (z) to look for segregation by sex in the data. Diving behaviours (maximum dive depth, dive duration and surface duration) were used as response variables in separate GAM analyses to investigate how habitat, FPT and seasonality (month) might explain diving strategies (referred to as “diving behaviour models”).

A GAM can deal with simple random effects, such as individual variability, by treating random effects as smooths [60]. This is implemented in the GAM by s(w, bs  =  "re") where w is the covariate of the smooth (here the individual seal id), bs is a basis penalty smoother, and the "re" class implements simple random effects [60]. The restricted/residual maximum likelihood estimation (REML) is used to correct for the degrees of freedom when there is an increased number of fixed parameters in the model [61]–[62]. It thereby produces an unbiased estimation of the variance parameters [61].

Bottom depth, TCI, maximum dive depth and surface duration were square root transformed and FPT was log transformed to obtain normal distribution. To select between competing models we applied an information-theoretic approach and examined parameter weightings using Akaike’s Information Criterion (AIC). All models (30 for the FPT habitat model and 56 for each of the diving behaviour models) nested within the full model were assumed to be candidate models. Models with ΔAIC < 2 are considered to have substantial support and ΔAIC >10 have very little support (ΔAIC is the difference between the AIC of the best fitting model and that of model *i*, [63]). If the addition of one predictor variable to a model resulted in an AIC of < two values from the model without this variable, and the model fit was not improved (deviance explained), the added variable was deemed a pretending variable and removed from the analysis [64].

The fitted values from the best habitat model (FPT as the response variable) were back-transformed before plotting a predictive surface using ArcGIS 10 (ESRI® ArcMap™ 10.0). Predicted graphs for each of the best models (both habitat and behavioural models) were created to present the fitted results for each of the predictor variables.

## Results

### Dive statistics across all seals

**Figure 2:**
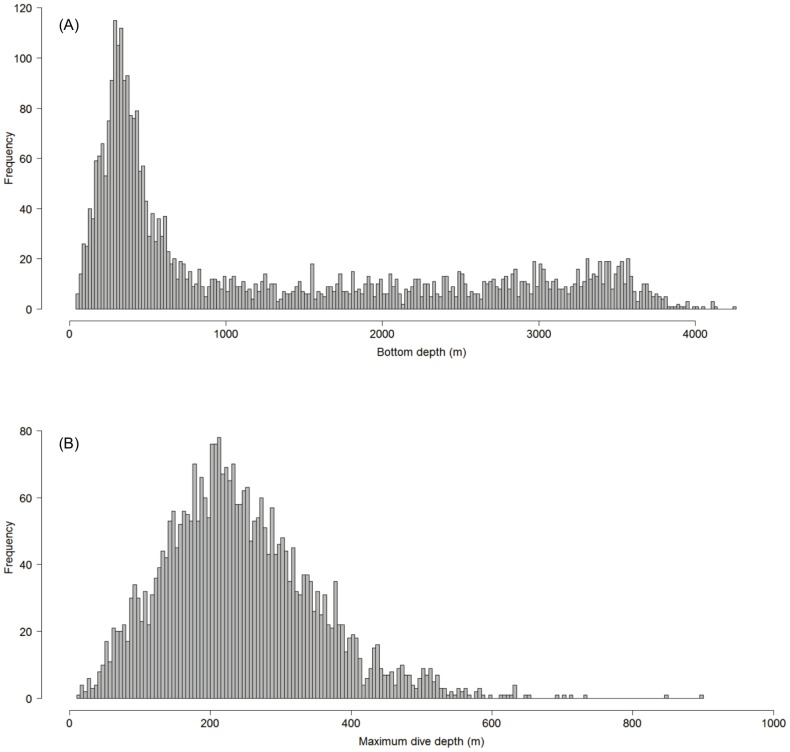
Frequency distributions. a) Frequency distribution of bottom depth across all seals (n  =  3,269) based on the number of dive locations obtained from the SRDL tags. b) Frequency distribution of maximum dive depth across all seals (n  =  3,269) based on number of dive locations obtained from the SRDL tags.

Of a total of 352,438 dive locations, the summary data, aggregated at the ARS scale of 40 km, yielded 3,269 data points. Dive statistics of the non-aggregated data are presented in Table 1. We found that 50% of the total number of dives recorded occurred in waters less than 500 m deep (Fig. 2a). In addition, 90% of the maximum dive depths did not exceed 500 m and 35% of the total number of dives had a maximum dive depth between 150−300 m (Fig. 2b). We also found that females used waters with bottom depths 30% deeper in the post-breeding/pre-moult period (April-June; F  =  5.742, p<0.05) and 40% deeper during the post-moult/pre-breeding period (August-February; F  =  6.804, p<0.05) than males.

**Table 1:** Dive statistics (non aggregated data) of dive behaviours (surface duration (SuD), dive duration (DD), maximum dive depth (MDD)) and habitat variables (bottom depth (BD), TCI) as well as FPT throughout the year (March and July are excluded) across all seals (n  =  352, 438).

	Sample size	SuD (min)	DD (min)	MDD (m)	BD (m)	TCI	FPT (h)
max all	352,438	9.45	57.25	1652.30	4293.0	22.80	952.60
mean all		1.8± (1.1)	13.9 ± (7.0)	255± (184.9)	1048.3± (1021.1)	7.5± (2.7)	167.3± (200.3)
max male	131,385	9.45	57.25	1652.30	3992.0	22.39	952.57
mean male		1.9± (1.1)	14.5± (7.6)	254.7± (196.6)	733.8± (774.7)	7.7± (2.7)	211.8± (251.2)
max female	221,053	9.45	39.25	1592.3	4293.0	22.80	718.08
mean female		1.7± (1.1)	13.5± (6.5)	255.1± (177.5)	1235.2± (1100.9)	7.5± (2.7)	140.8± (156.5)

Mean is given with ±SD in parentheses.

Spearman correlation statistics were run on the aggregated data between dive behaviours and bottom depth (Table 2). The relationship between the variables was significant, but the levels of linear correlation was relatively low (df  =  12 r_s_  =  0.05−0.25) except for a stronger positive correlation between maximum dive depth and dive duration (df  =  12, r_s_  =  0.58, Table 2).

**Table 2:** Spearman rank correlation table presenting the relationship between the surface duration (SuD), dive duration (DD), maximum dive depth (MDD) and bottom depth (BD) across all seals (n  =  3,269).

	SuD	DD	MDD	BD
**SuD**		0.25	0.36	0.15
**DD**	0.25		0.58	0.05
**MDD**	0.36	0.58		0.36
**BD**	0.15	0.05	0.36	

All r_s_ in the table are significant at p < 0.05.

### Habitat model: Segregation by sex in relation to FPT

**Figure 3:**
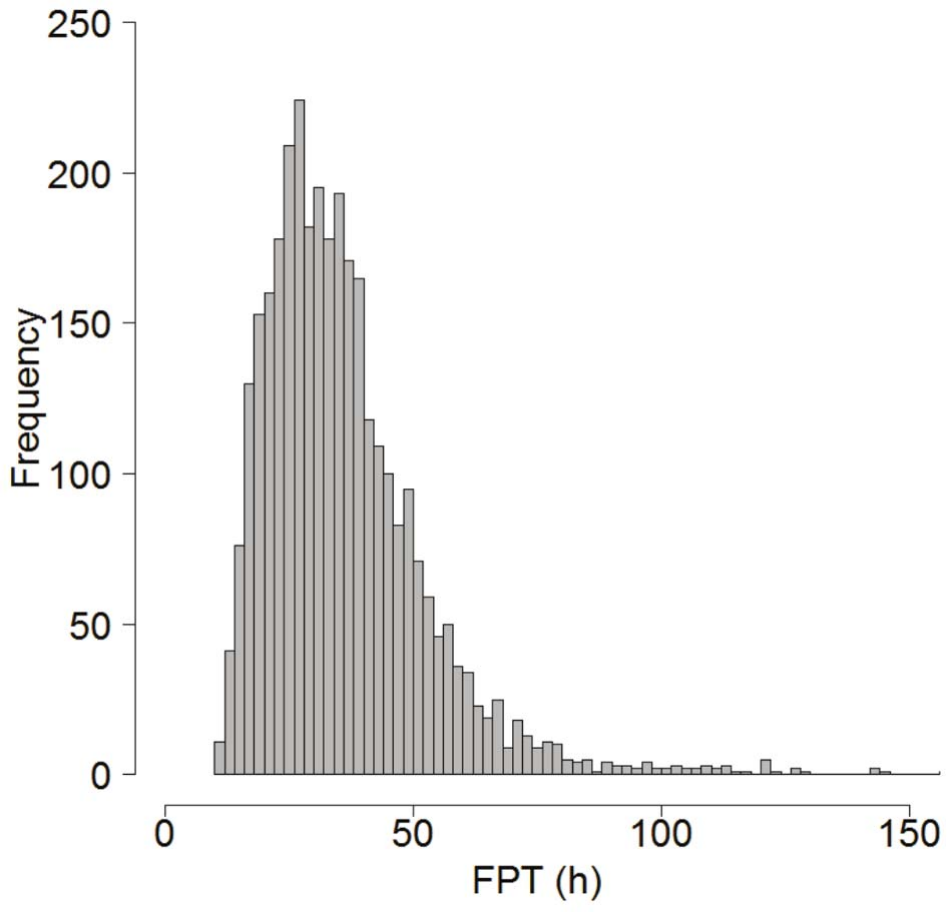
Histogram showing the fitted distribution of FPT across all seals (n  =  3,269) from our best habitat model.

**Figure 4:**
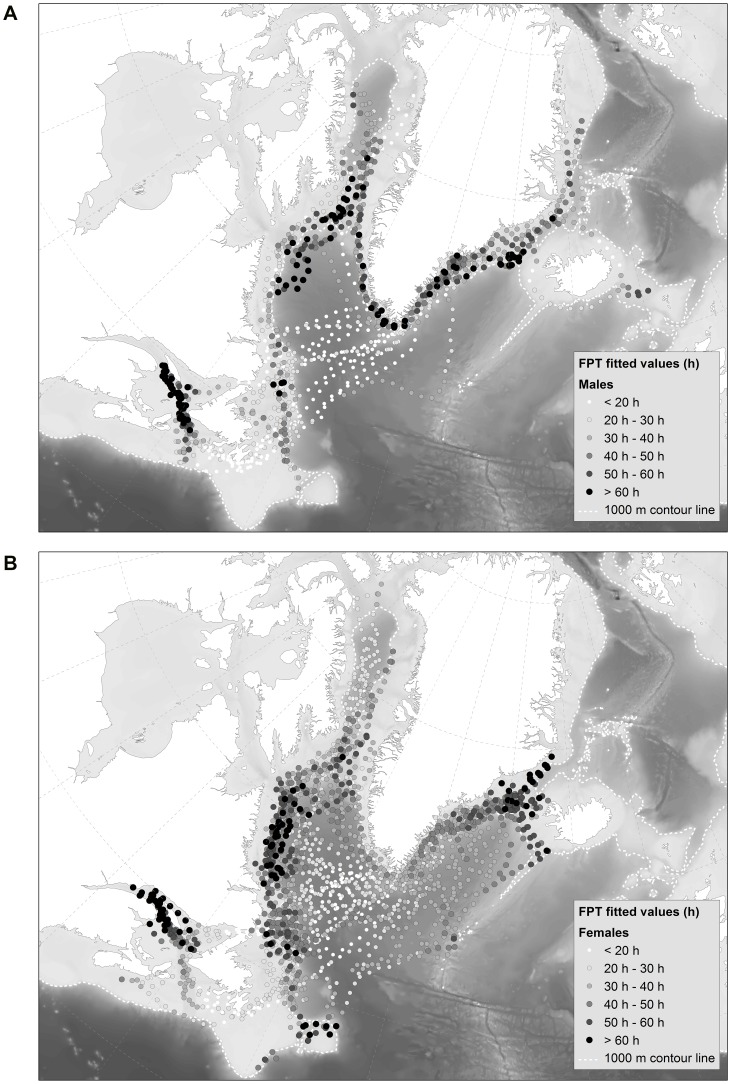
Fitted values from the best habitat model (Geographic location, Month, Bottom depth) for a) males (n = 18) and b) females (n = 33). Track is plotted on the 80 km ARS scale. Filled circles represent FPT from < 20 hours − > 60 hours (light to dark). The dashed white line is the 1,000 m contour line and the bathymetry is represented by the grey scale backdrop.

**Figure 5:**
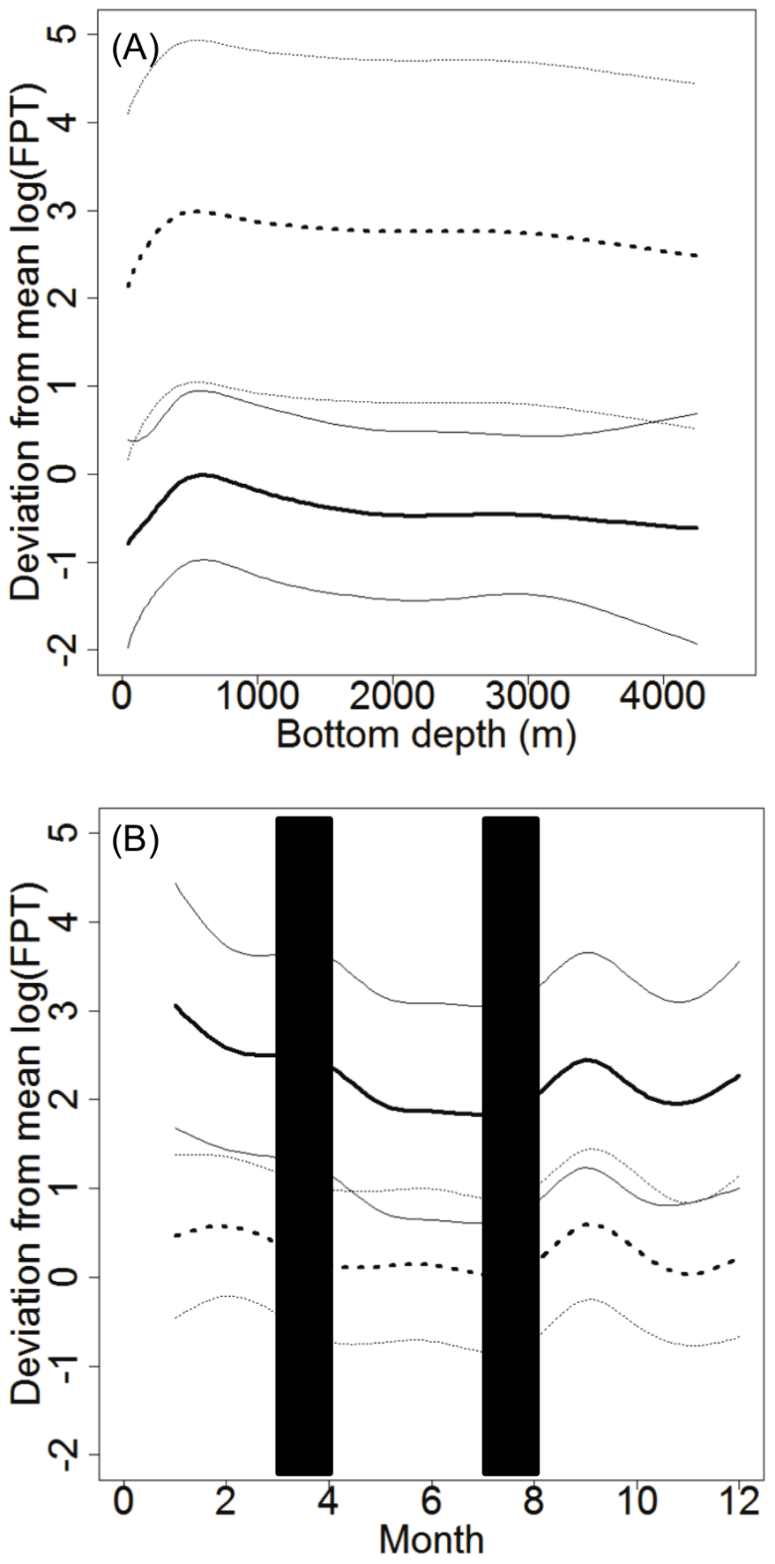
Habitat model results. Predicted results for a) Bottom depth (m) and b) Month (blue columns represent approximate fasting periods) for male (n = 18) and female (n = 33) hooded seals. Solid black line represents males and hashed line represents females. Thin black lines represent the standard error.

The GAM analysis of habitat relationships indicated that hooded seals were segregated by sex in relation to geographic location, month and bottom depth in terms of FPT (*w_i_*  =  0.833, Table 3). The deviance explained was 31.4%. The next best model included the following predictor variables: geographic location, month, bottom depth and TCI by sex ((ΔAIC  =  3.2, *w_i_*  =  0.168, Table 3). The deviance explained for this model was also 31.4%, suggesting that TCI was acting as a pretending variable. All other models had a ΔAIC > 10 and were highly implausible (Table S1). The map created on the back-transformed fitted values from the best habitat model (frequency histogram presented in Fig. 3) showed that males exhibited long FPTs along the shelf areas, especially the northern parts of the Labrador shelf and in Davis Strait (Fig. 4a). Females had long FPTs mainly along the Labrador shelf and were also distributed over deeper waters than males (Fig. 4b, Fig. 5a). Some seals spent extended time in SE Greenland and the Denmark Strait area, the Front, Flemish Cap and in the Gulf of St. Lawrence (Gulf breeding animals only, Fig. 4).

**Table 3:** AIC table presenting the best models.

RV	P	loglik	K	AIC_i_	ΔAIC	exp(−0.5Δ_i_)	w_i_	DE (%)
FPT	GL, BD and M by sex	−3293.69	7	66601.39	0	1	0.8323	31.4
	GL, BD, TCI and M by sex	−3293.30	9	6604.59	3.20	0.2014	0.1677	31.4
MDD	GL, FPT, BD, TCI and M by sex	−7380.97	11	14783.94	0	1	0.9966	57
DD	GL, FPT, BD, TCI and M by sex	−21741.35	11	43504.7	0	1	0.7925	65.3
	GL, FPT, BD and M by sex	−21744.69	9	43507.38	2.68	0.2618	0.2075	65.3
SuD	GL, FPT, BD, TCI and M by sex	−4488.28	11	8998.57	0	1	0.6599	39.8
	GL, FPT, BD and M by sex	−4490.95	9	8999.9	1.326	0.5153	0.3401	39.7

The response variables (RV; FPT, maximum dive depth (MDD), dive duration (DD, surface duration (SuD)) were investigated in relation to geographic location (GL), bottom depth (BD), month (M) and TCI. The behavioural models included FPT as a predictor variable (P). Loglik is the loglikelihood, K is the number of parameters in the model. AIC_i_ is AIC for model i, and ΔAIC is the difference between the AIC of the best fitting model and that of model i. Exp(−0.5Δ_i_) represent the relative likelihoods and the w_i_ is the Akiake weight. D.E (%) is the deviance explained by the model. Tables showing all candidate models are presented in the supplementary material (Tables S1−S4).

The predicted results demonstrated that seals spent most of their time in waters of approximately 700 m bottom depth, exhibiting a decrease in passage time (shorter FPT) across shallower and deeper waters (Fig. 5a). Females spent longer time periods across all bottom depths than males (Fig. 5a). FPT was longer in September during the post-moult period and through the winter during the pre-breeding period (December-February) for both sexes (Fig. 5b).

### Behavioural models: Segregation by sex in relation to diving behaviour

**Figure 6:**
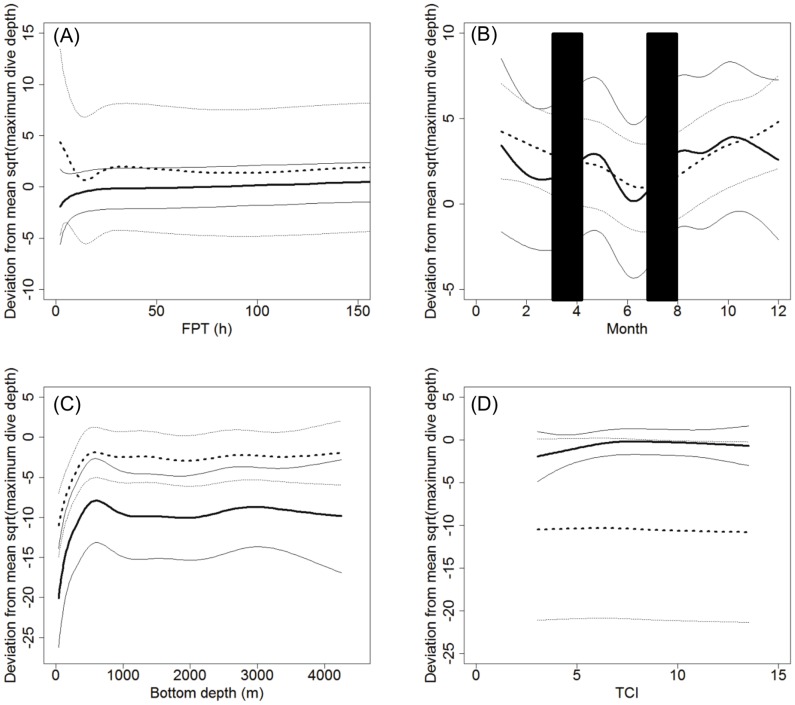
Maximum dive depth model results. Predicted results for a) FPT (h), b) Month (blue columns represent approximate fasting periods), c) Bottom depth (m) and d) TCI for male (n = 18) and female (n = 33) hooded seals. Solid black line represents males and hashed line represents females. Thin black lines represent standard error.

**Maximum dive depth.** The GAM analysis of diving depth relationships had the highest support for the full model (geographic location, FPT, bottom depth, month and TCI) including a separation by sex per predictor variable (*w_i_*  =  0.9966, Table 3). The deviance explained was 57%. All other candidate models had a ΔAIC > 10 (Table S2). Males and females exhibit similar patterns of fluctuations in terms of maximum diving behaviour, except in relation to TCI (Fig. 6d). Males increased dive depths towards areas where they spent >20 h while females initially decreased dive depth from 0−20 FPT prior to increasing dive depth towards areas where they spent >30 hours. Maximum dive depth then stabilised across longer FPT for both sexes (Fig. 6a, Fig. S1). The standard error was larger for females, and overlapped completely with that of males, which indicates a considerable amount of variability among females (Fig. 6a).

The maximum dive depth fluctuated across all months of the year and this behaviour was very similar between males and females. However, males displayed shallower dive depths directly before breeding (March) and moulting (July) (Fig. 6b). Dive depths increased following these periods reaching a peak in May and then again from August to November (Fig. 6b). Females underwent their deepest dives during the winter (December/January) with lower values prior to the moult (Fig. 6b).

Both males and females increased their maximum dive depths from bottom depths of 0−600 m (Fig. 6c). They appeared to reduce their dive depths as bottom depth increased from ∼600 to 1,000 m, but both sexes stabilised their dive depth across all bottom depths of >1000 m (Fig. 6c).

Female dive depths did not seem to be influenced by TCI, while males showed a slight positive relationship to a TCI value of ∼7, above which no relationship to TCI was detected (Fig. 6d). The standard error was large for females, which means that there was considerable variability related to this variable.

**Dive duration.** The GAM analysis of dive duration relationships had the highest support for the full model (geographic location, FPT, bottom depth, month and TCI) including a separation by sex per predictor variable (*w_i_*  =  0.7925, Table 3). The deviance explained was 65.3%. The next best model included the same predictors except TCI and had a ΔAIC of 2.68, making this model less plausible (*w_i_*  =  0.2075). However, the deviance explained was the same as the best model (65.3%), suggesting that TCI was acting as a pretending variable. All other candidate models had ΔAIC > 10 (Table S3).

**Figure 7:**
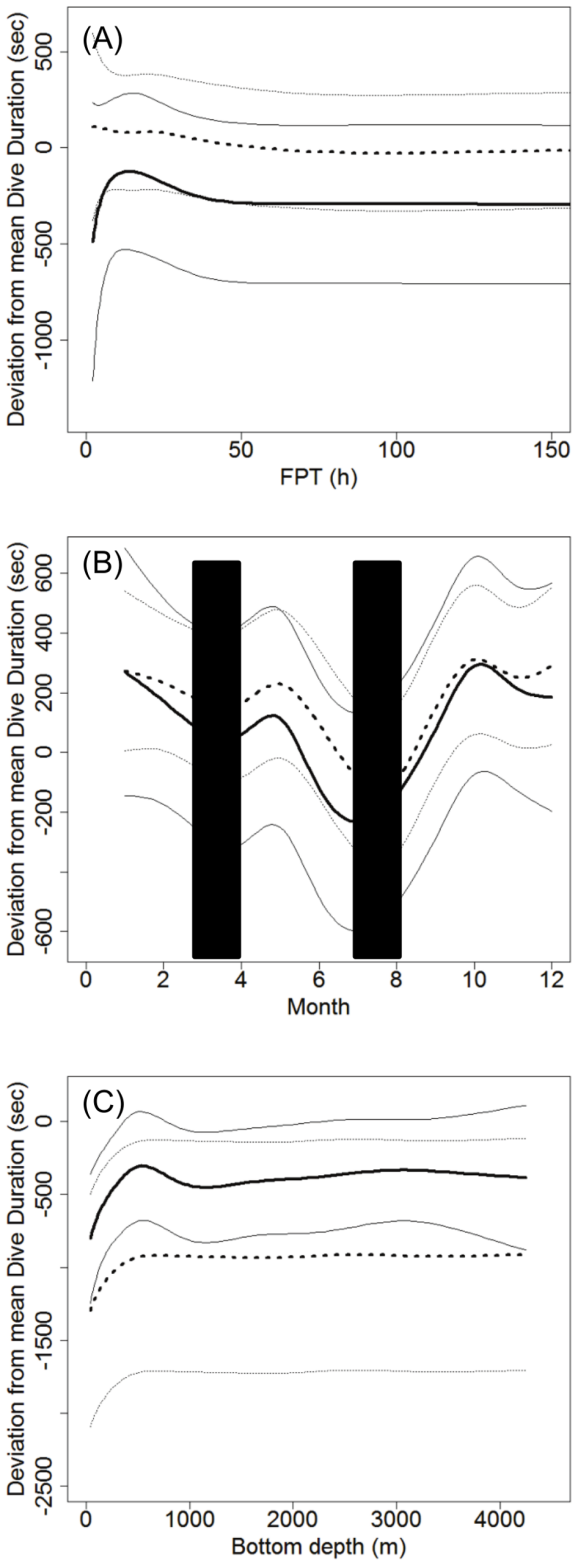
Dive duration model results. Predicted results for a) FPT (h), b) Month (blue columns represent approximate fasting periods) and c) Bottom depth (m) for male (n = 18) and female (n = 33) hooded seals. Solid black line represents males and hashed line represents females. Thin black lines represent standard error.

Males exhibited a decrease in dive duration from 20 − 50 hour FPTs and no relationship between dive duration and FPT with longer passage times was observed for either sex (Fig. 7a, Fig. S2). Both sexes showed variable dive durations across months. The model predicted an increase in May and decrease towards the moult in July (Fig. 7b). Following the moult, dive duration increased with a peak in October and January (Fig. 7b).

Dive duration peaked for both sexes at bottom depths of 600 to 800 m (Fig. 7c). Males exhibited a decrease in dive durations as bottom depths increased to 1,000 m, before they increased again toward 3,000 m depth, while dive durations did not change for females across deeper depths (Fig. 7c).

**Surface duration.** The GAM analysis of surface duration relationships had the highest support for the full model (geographic location, FPT, bottom depth, month and TCI) including a separation by sex per predictor variable (*w_i_*  =  0.6599, Table 3). The deviance explained was 39.8%. The next best model did not include TCI and had a ΔAIC of 1.33, making this model highly plausible (*w_i_*  =  0.3401, Table S3). The deviance explained was 39.7%, which again suggests that TCI acted as a pretending variable. All other candidate models had a ΔAIC > 10 (Table S4).

**Figure 8:**
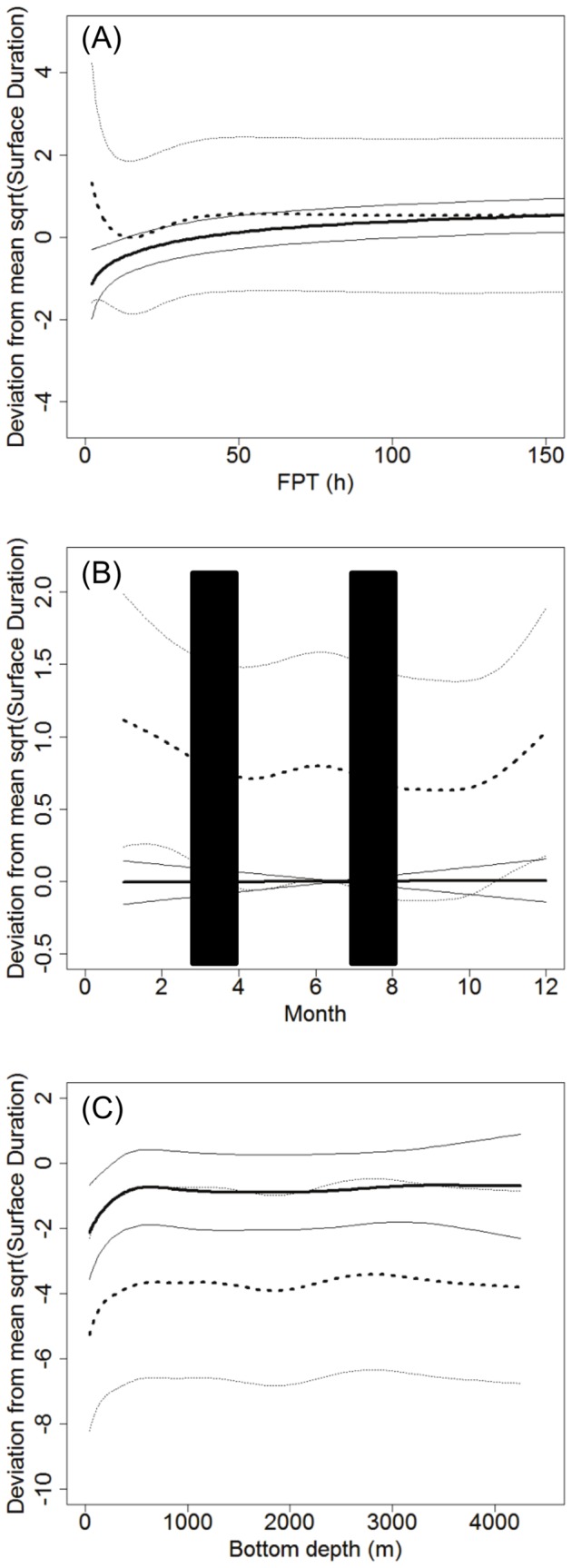
Surface duration model results. Predicted results for a) FPT (h), b) Month (blue columns represent approximate fasting periods) and c) Bottom depth (m) for male (n = 18) and female (n = 33) hooded seals. Solid black line represents males and hashed line represents females. Thin black lines represent standard error.

Although there is considerable variation among females, they displayed an increase in surface duration with increasing FPT up to approximately 50 h, while males continued to increase the time spent at the surface between dives up to approximately 150 h FPT from when it started to stabilise (Fig. 8a, Fig. S3).

There was no relationship between surface duration and season in males. Females had longer surface durations during late winter, early spring and around the moult, although there was a high amount of variability in the data (Fig. 8b). Both males and females increased surface durations with increasing bottom depths to ∼600 m (Fig. 8c). Males then had the same surface duration across all bottom depths, while females showed some fluctuation across deeper bottom depths (Fig. 8c).

## Discussion

Hooded seals have the highest capability to store oxygen in blood and skeletal muscles measured for any pinniped [65]. The seals in this study exhibited a maximum dive depth of 1,652.3 m for a male, and 1,592.3 m for a female, and maximum dive duration of 57.25 min (Table 1). Only males demonstrated a dive duration of more than 39.25 min, however, Folkow and Blix [44] observed that females from the Greenland Sea population could dive for >52 min. Among the pinnipeds, this diving ability can probably only be matched by elephant seals (e.g., [36], ). For much of the year, hooded seals appear to utilise (i.e., have the longest passage time through) areas with mean bottom depths between 700−1,200 m (Fig. 5a, Table 1). In these areas they carry out dives into the mesopelagic zone (annual mean maximum dive depth was 255 ± 184.9 m), along the shelf break areas and areas of high topographic relief. These results are similar to what has been observed among the Greenland Sea population of hooded seals, which were found to regularly dive to depths between 100–600 m, although the dive depths varied between areas and seasons [44]. Folkow and Blix [44] also estimated the average dive durations for the Greenland Sea hooded seals to be 5−15 min, while seals in our study had mean dive durations at the high end of their range (13.5−14.5 min, Table 1). These differences are likely due to the fact that the data available to Folkow and Blix [44] was binned into predefined periods, which did not allow for fine scale observations.

In this study, we demonstrated that hooded seals are segregated by sex in terms of FPT and diving behaviours. Our best habitat model identified geographic location, bottom depth and seasonality as the most important explanatory variables. Habitat selection was not influenced by TCI, which is consistent with the findings by Andersen et al. [4]. They found a low level of importance by slope when this was applied as a measure of bathymetry in their habitat model. These findings suggest that bathymetry alone may not be a good explanatory variable in terms of habitat selection and the time hooded seals spend in a particular location. In fact, our findings suggest that bathymetry acts as a pretending variable, which means that it does not improve the fit of the model [64]. The complex topographic properties may serve as a weak proxy for other oceanographic processes, such as movements of currents, and thereby temperature and productivity at depth, which might be of higher importance.

Another parameter that is likely to be important, and the distribution of which is driven by oceanographic processes, is the measure of prey availability. Prey availability is hard to measure in the marine environment (e.g., [68]) and more so in the high Arctic where suitable abundance estimates are limited, especially for non-commercially important species. Thus prey could not be included in the models here. However, some spatial inferences have been made from diet data. Hammill and Stenson [13] found geographic variation in dominant prey species in the diet depending on sampling area. They also found that, although hooded seals forage on benthic species such as Greenland halibut, redfish and Atlantic cod, most of the prey present in the stomachs consisted of juveniles, which are often found higher in the water column than adult fish (e.g., [69]). This suggests that hooded seals carry out pelagic feeding dives within their high use areas as mean dive depths were 255±184.9 m and mean bottom depths ranged from 700−1,200 m. Only 10% of the dives were deeper than 500 m. However, as hooded seals appear to be generalist feeders with a high diversity of prey species in their diet (e.g., [13], ), real time inferences about their diet requires spatial and temporal overlap in sampling of both diet data (or at the least observed predator presence) and prey presence (including abundance estimates).

GAM models that include an indicator variable (sex) were found to be the best models, thereby identifying sexual segregation in the data. A significant statistical difference in habitat selection and diving behaviour between male and female hooded seals was observed, but the diving behaviour models were described with considerable overlap of predicted results. This could be due to behavioural variation of the individuals or variability in terms of body size and diving capabilities. However, our results suggest that overall diving behaviour is influenced by FPT, geographic location, bottom depth and season. Similarly to the results from the habitat model, the behavioural models suggest that the surface duration and dive duration was not influenced by TCI. However, the model identified TCI as an important predictor of maximum dive depth. We observed a weak positive relationship with TCI by males to a value of ∼7 (mean  =  7.7 ± 2.7, Table 1). TCI for the entire study area ranges from 0.5−25, where a high number represents peaks and a low number represents basins. This suggests that male hooded seals adjust their dive depth according to topography, representing a “downward slope” toward basins more than an “upward slope” toward peaks (TCI of ∼12.5 represents a flat surface). Such areas could, for instance, describe the shelf break where males spend extended periods of time at various times of the year. Females’ exhibited no (or slightly negative) relationship between dive depth and TCI, and the results were highly variable. This lack of relationship could be linked to females preferring deeper water than males, where prey distribution may not be as affected by the topographic complexity of the sea floor as over the shelf [70].

Both migration periods are characterised by an increase in dive duration for both sexes (peak in May, October and January). These increases could represent periods of higher foraging intensity when the seals gain body mass and energy reserves after fasting periods in preparation for the next fasting period. Males also demonstrated an increase in maximum dive depth during these periods, but did not demonstrate a pronounced change in surface durations. This may be explained by the hooded seal’s high capability to store oxygen and recover quickly from diving [65]. Females had a less pronounced variability in dive depth throughout the year, but the deepest diving occurred during the winter months (December-January), and the surface duration seemed to fluctuate similarly to dive depth (Fig. 6b, Fig. 8b). There was a slight increase in surface duration from May with a peak in June prior to the moult, which is consistent with the increase in dive duration and a small increase in dive depth during the same time period. Females also demonstrated an increase in surface duration over the winter months, and although these results are represented with some variability, the combined diving behaviour and longer FPT in between fasting periods suggest that the smaller females, especially if carrying a foetus, may have a greater need to recover from dives than that of the larger males. These findings differ somewhat from what has been found for the Greenland Sea hooded seal stock. Folkow and Blix [44] observed no sexual segregation (4 males and 12 females), and the deepest and longest dives occurred in the winter months and not necessarily in relation to fasting periods as observed for the NW Atlantic stock. A difference in foraging behaviour between the two stocks may be explained by the differences in the environmental properties of their range.

Little has been known previously about hooded seal sexual segregation during the post-moult/pre-breeding period (August-February), although Bajzak et al. [5] found sexual segregation by hooded seals in the Gulf of St. Lawrence during the post-breeding/pre-moult period (April-June). They found that females dived, on average, 70 m shallower than males prior to migration, and 40 m deeper than males following migration, demonstrating vertical, but not horizontal segregation [5]. We found that males and females were spatially segregated in relation to bottom depth (Fig. 5a), where females used areas with bottom depths 30% deeper post-breeding/pre-moult and 40% deeper during post-moult/pre-breeding than males, but no significant difference in dive depth was observed. The differences in the degree of spatial overlap within the Gulf breeding animals and within the Front (and Davis Strait) breeding animals during parts of the migration is probably due to the small numbers of the Gulf breeding herd and spatial limitations of the Laurentian Channel (∼500 m deep, Fig. 1) compared to the areas available for Front breeding seals [5]. The Front breeding herd comprises 90% of the NW Atlantic population which means that they may need to spread out more to access suitable feeding areas [71]. Thus, the Gulf may be able to sustain both males and females of the small herd residing there during the post-breeding period, but they segregate vertically instead of geographically. During the pre-moult period the segregation behaviour becomes more similar to the rest of the population.

Females occupying waters with deeper bottom depths than males may be targeting vertically migrating benthopelagic prey with diurnal cycles across deeper depths than species occupying less deep waters. Folkow and Blix [44] found that the Greenland Sea hooded seal stock exhibited similar diurnal variations in diving depths implying foraging on diurnally migrating prey, and there was no difference between males and females. Le Boeuf et al. [21] found a difference in distribution patterns of northern elephant seals, where females carried out consistent pelagic foraging while venturing across a broad expanse of the northeast Pacific Ocean, and males would target the continental margin. They suggested that males fed on food sources determined by spatial boundaries, while females were utilising a food source determined by a fixed cyclical pattern of vertical prey movement in the pelagic and mesopelagic environment. Diets of NW Atlantic hooded seals differ between males and females, even within seasons [35] and a difference in prey selection could account for the differences in habitat locations we observed, or the difference in habitat locations could explain differences in prey items available. Our findings are similar to those of Le Boeuf et al. [21] in that male hooded seals have longer FPTs according to month than females (Fig. 5b), which suggests that males spend more time in profitable areas while females travel with shorter FPT, covering a larger area (hence more time spent over all bottom depths; Fig. 5a).

Spatial segregation and different diving behaviours between males and females could indicate that the energy requirements (and hence dietary needs) are different due to different energy expenditure during fasting periods (e.g., during the breeding period males loose ∼14% of their mean body mass over ∼2.5 weeks [24], while females loose ∼16% mean body mass over 4 days [25]−[26]). A horizontal spatial segregation between sexes may have evolved as a result of a resource partitioning strategy to avoid sexual competition.

In summary, we have demonstrated that male and female hooded seals in the North Atlantic Ocean are spatially segregated in relation to the selection of habitat throughout the annual migration and by diving behaviour in relation to FPT, bathymetry and life history events such as whelping/breeding and moulting periods. According to the habitat model, NW Atlantic hooded seals have the highest dive frequency in areas where the FPT is approximately 20−40 h (Fig. 3) and this is also where they start to carry out deeper diving and exhibit longer dive durations. There is also a slight increase in surface duration during this time of restricted search and assumed foraging. These findings suggest that diving behaviour change with passage time according to the oceanographic properties of the ARS area, and that males and females differ in respect to these properties. This information offers valuable knowledge of the habitat selection of male and female hooded seals in the NW Atlantic Ocean. Changes in body condition associated with these areas of high use may confirm their importance as feeding areas, which can further improve our understanding of the ecology of the species.

## Supporting Information

Figure S1 **Full predicted maximum dive depth model results for FPT as seen in** **Figure 6a** **across all hours of FPT.** Solid black line represent males (n = 18) and the hashed line represent females (n = 33). Thin black lines represent the standard error.
(TIF)

Figure S2 **Full predicted dive duration model results for FPT as seen in** **Figure 7a** **across all hours of FPT.** Solid black line represent males (n = 18) and the hashed line represent females (n = 33). Thin black lines represent the standard error.
(TIF)

Figure S3 **Full predicted surface duration model results for FPT as seen in** **Figure 8a** **across all hours of FPT.** Solid black line represent males (n = 18) and the hashed line represent females (n = 33). Thin black lines represent the standard error.
(TIF)

Table S1 **AIC table presenting all candidate GAM models with FPT as a response variable.** The response variable was investigated in relation to geographic location (GL), bottom depth (BD), month (M) and TCI. Loglik is the loglikelihood, K is the number of parameters in the model. AIC_i_ is AIC for model *i*, and ΔAIC is the difference between the AIC of the best fitting model and that of model *i*. Exp(−0.5Δ_i_) represent the relative likelihoods and the *w*_i_ is the Akiake weights. D.E% is the deviance explained by the model.
(DOC)

Table S2 **AIC table presenting all candidate GAM models with maximum dive depth as a response variable.** The response variable was investigated in relation to geographic location (GL), FPT, bottom depth (BD), month (M) and TCI. The behavioural models included FPT as a predictor variable. Loglik is the loglikelihood, K is the number of parameters in the model. AIC_i_ is AIC for model *i*, and ΔAIC is the difference between the AIC of the best fitting model and that of model *i*. Exp(−0.5Δ_i_) represent the relative likelihoods and the *w*_i_ is the Akiake weights. D.E% is the deviance explained by the model.
(DOC)

Table S3 **AIC table presenting all candidate GAM models with dive duration as a response variable.** The response variable was investigated in relation to geographic location (GL), FPT, bottom depth (BD), month (M) and TCI. The behavioural models included FPT as a predictor variable. Loglik is the loglikelihood, K is the number of parameters in the model. AIC_i_ is AIC for model *i*, and ΔAIC is the difference between the AIC of the best fitting model and that of model *i*. Exp(−0.5Δ_i_) represent the relative likelihoods and the *w*_i_ is the Akiake weights. D.E% is the deviance explained by the model.
(DOC)

Table S4 **AIC table presenting all candidate GAM models with surface duration as a response variable.** The response variable was investigated in relation to geographic location (GL), FPT, bottom depth (BD), month (M) and TCI. The behavioural models included FPT as a predictor variable. Loglik is the loglikelihood, K is the number of parameters in the model. AIC_i_ is AIC for model *i*, and ΔAIC is the difference between the AIC of the best fitting model and that of model *i*. Exp(−0.5Δ_i_) represent the relative likelihoods and the *w*_i_ is the Akiake weights. D.E% is the deviance explained by the model.
(DOC)
