# Supplementary figures and images for: Investigating Annual Diving Behaviour by Hooded Seals (Cystophora cristata) within the Northwest Atlantic Ocean
Source: PLoS One. 2013 Nov 25;8(11):e80438. doi: 10.1371/journal.pone.0080438 (PMC3840026; doi:10.1371/journal.pone.0080438)

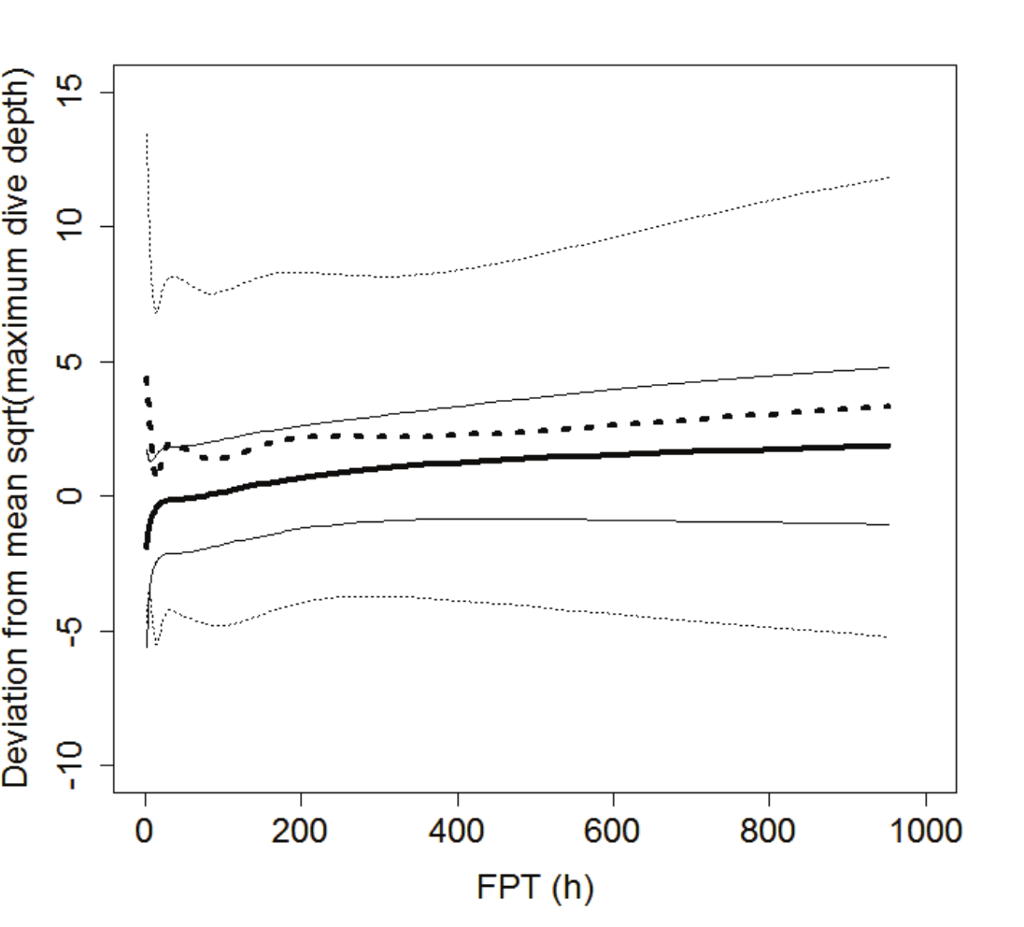

Supplement: Figure S1 — Full predicted maximum dive depth model results for FPT as seen in Figure 6a across all hours of FPT. Solid black line represent males (n = 18) and the hashed line represent females (n = 33). Thin black lines represent the standard error. (TIF) [file pone.0080438.s001.tif]

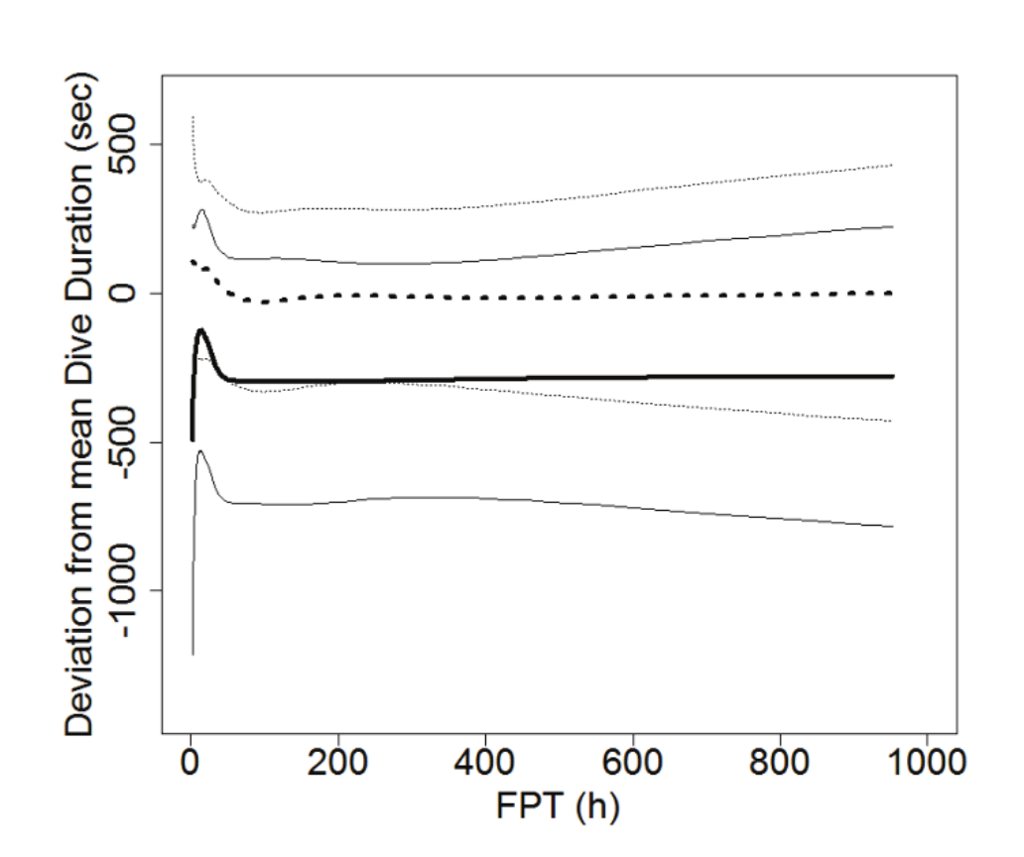

Supplement: Figure S2 — Full predicted dive duration model results for FPT as seen in Figure 7a across all hours of FPT. Solid black line represent males (n = 18) and the hashed line represent females (n = 33). Thin black lines represent the standard error. (TIF) [file pone.0080438.s002.tif]

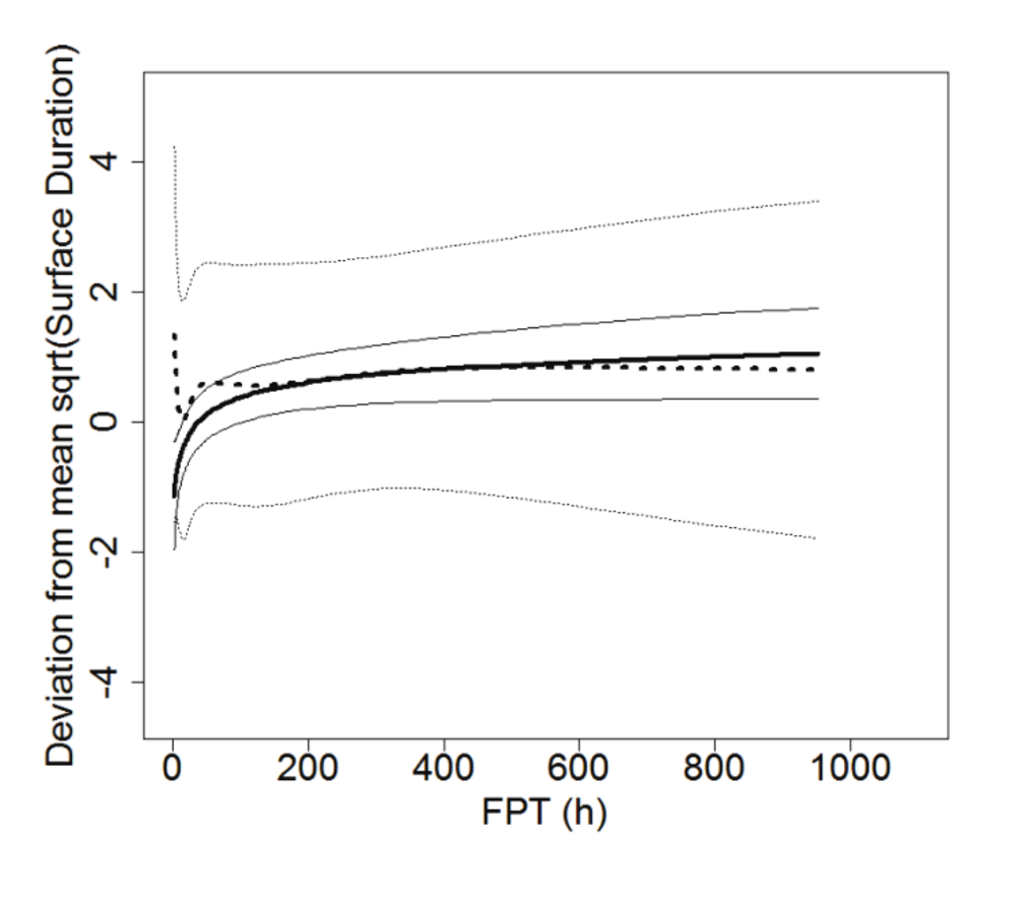

Supplement: Figure S3 — Full predicted surface duration model results for FPT as seen in Figure 8a across all hours of FPT. Solid black line represent males (n = 18) and the hashed line represent females (n = 33). Thin black lines represent the standard error. (TIF) [file pone.0080438.s003.tif]
