# Supplementary material for: Investigating Annual Diving Behaviour by Hooded Seals (Cystophora cristata) within the Northwest Atlantic Ocean
Source: PLoS One. 2013 Nov 25;8(11):e80438. doi: 10.1371/journal.pone.0080438 (PMC3840026; doi:10.1371/journal.pone.0080438)
Supplement: Table S1 — AIC table presenting all candidate GAM models with FPT as a response variable. The response variable was investigated in relation to geographic location (GL), bottom depth (BD), month (M) and TCI. Loglik is the loglikelihood, K is the number of parameters in the model. AICi is AIC for model i, and ΔAIC is the difference between the AIC of the best fitting model and that of model i. Exp(−0.5Δi) represent the relative likelihoods and the w i is the Akiake weights. D.E% is the deviance explained by the model. (DOC) [file pone.0080438.s004.doc]

Table S1:

| model | loglik | K | AIC_i_ | ΔAIC | exp(-0.5Δ_i_) | *w_i_* | DE (%) |
| --- | --- | --- | --- | --- | --- | --- | --- |
| GL, BD and M by sex | -3293.69 | 7 | 6601.39 | 0.00 | 1 | 0.833 | 31.4 |
| GL, BD, TCI and M by sex | -3293.30 | 9 | 6604.59 | 3.20 | 0.20149 | 0.168 | 31.4 |
| GL, BD, TCI and M | -3349.04 | 5 | 6708.08 | 106.69 | 6.8E-24 | 5.64899E-24 | 29 |
| GL, BD and M | -3350.45 | 4 | 6708.90 | 107.51 | 4.5E-24 | 3.75646E-24 | 28.9 |
| GL and M by sex | -3359.54 | 5 | 6729.07 | 127.68 | 1.9E-28 | 1.56333E-28 | 28.5 |
| GL, BD and TCI by sex | -3372.67 | 7 | 6759.33 | 157.94 | 5.1E-35 | 4.20348E-35 | 28 |
| GL and BD by sex | -3379.22 | 5 | 6768.44 | 167.05 | 5.3E-37 | 4.4286E-37 | 27.7 |
| GL ,TCI and M by sex | -3390.54 | 7 | 6795.07 | 193.69 | 8.7E-43 | 7.27609E-43 | 27.2 |
| GL ,BD and TCI | -3413.72 | 4 | 6835.44 | 234.05 | 1.5E-51 | 1.24891E-51 | 26.1 |
| GL and BD | -3416.41 | 3 | 6838.81 | 237.43 | 2.8E-52 | 2.31141E-52 | 26 |
| GL and M | -3421.33 | 3 | 6848.66 | 247.27 | 2E-54 | 1.68208E-54 | 25.8 |
| GL, TCI and M | -3420.47 | 4 | 6848.95 | 247.56 | 1.7E-54 | 1.45649E-54 | 25.8 |
| BD and M by sex | -3439.15 | 5 | 6888.29 | 286.91 | 5E-63 | 4.16325E-63 | 25 |
| GL by sex | -3450.60 | 3 | 6907.20 | 305.81 | 3.9E-67 | 3.26623E-67 | 24.5 |
| GL and TCI by sex | -3449.33 | 5 | 6908.67 | 307.28 | 1.9E-67 | 1.56931E-67 | 24.5 |
| BD, TCI and M by sex | -3450.78 | 7 | 6915.56 | 314.17 | 6E-69 | 5.00184E-69 | 24.4 |
| BD, TCI and M | -3464.51 | 4 | 6937.02 | 335.63 | 1.3E-73 | 1.09433E-73 | 23.8 |
| BD and M | -3465.86 | 3 | 6937.72 | 336.33 | 9.2E-74 | 7.69624E-74 | 23.7 |
| GL and TCI | -3490.45 | 3 | 6986.90 | 385.51 | 1.9E-84 | 1.61217E-84 | 22.6 |
| GL | -3491.84 | 2 | 6987.69 | 386.30 | 1.3E-84 | 1.08826E-84 | 22.5 |
| BD and TCI by sex | -3554.92 | 5 | 7119.83 | 518.44 | 3E-113 | 2.198E-113 | 19.5 |
| BD by sex | -3563.63 | 3 | 7133.27 | 531.88 | 3E-116 | 2.6546E-116 | 19 |
| BD and TCI | -3574.81 | 3 | 7155.62 | 554.23 | 4E-121 | 3.7145E-121 | 18.5 |
| BD | -3575.82 | 2 | 7155.64 | 554.25 | 4E-121 | 3.6849E-121 | 18.4 |
| M by sex | -3597.63 | 3 | 7201.27 | 599.88 | 5E-131 | 4.5498E-131 | 17.3 |
| M | -3620.40 | 2 | 7244.80 | 643.41 | 2E-140 | 1.6038E-140 | 16.2 |
| M and TCI | -3620.50 | 3 | 7246.99 | 645.61 | 6E-141 | 5.3545E-141 | 16.2 |
| M and TCI by sex | -3667.61 | 5 | 7345.21 | 743.82 | 3E-162 | 2.5174E-162 | 13.7 |
| TCI by sex | -3723.70 | 3 | 7453.41 | 852.02 | 1E-185 | 8.0709E-186 | 10.7 |
| <TCI> | -3728.67 | 2 | 7461.33 | 859.95 | 2E-187 | 1.5324E-187 | 10.4 |
