# Supplementary material for: Investigating Annual Diving Behaviour by Hooded Seals (Cystophora cristata) within the Northwest Atlantic Ocean
Source: PLoS One. 2013 Nov 25;8(11):e80438. doi: 10.1371/journal.pone.0080438 (PMC3840026; doi:10.1371/journal.pone.0080438)
Supplement: Table S2 — AIC table presenting all candidate GAM models with maximum dive depth as a response variable. The response variable was investigated in relation to geographic location (GL), FPT, bottom depth (BD), month (M) and TCI. The behavioural models included FPT as a predictor variable. Loglik is the loglikelihood, K is the number of parameters in the model. AICi is AIC for model i, and ΔAIC is the difference between the AIC of the best fitting model and that of model i. Exp(−0.5Δi) represent the relative likelihoods and the w i is the Akiake weights. D.E% is the deviance explained by the model. (DOC) [file pone.0080438.s005.doc]

Table S2:

| model | loglik | K | AIC_i_ | ΔAIC | exp(-0.5Δ_i_) | *w_i_* | DE (%) |
| --- | --- | --- | --- | --- | --- | --- | --- |
| GL, FPT, BD, TCI and M by sex | -7380.97 | 11 | 14783.94 | 0 | 1 | 0.996557126 | 57 |
| GL, FPT, BD and M by sex | -7388.64 | 9 | 14795.27 | 11.336 | 0.003454768 | 0.003442874 | 56.8 |
| GL, BD AND M by sex | -7434.44 | 7 | 14882.88 | 98.946 | 3.267E-22 | 3.25575E-22 | 55.6 |
| GL, FPT, BD, TCI and M | -7480.61 | 6 | 14973.22 | 189.28 | 7.91353E-42 | 7.88628E-42 | 54.3 |
| GL, FPT, BD and M | -7485.01 | 5 | 14980.01 | 196.074 | 2.64894E-43 | 2.63982E-43 | 54.2 |
| GL, FPT, BD and TCI by sex | -7511.15 | 9 | 15040.29 | 256.354 | 2.15495E-56 | 2.14753E-56 | 53.5 |
| GL, FPT and BD by sex | -7515.65 | 7 | 15045.29 | 261.356 | 1.76712E-57 | 1.76103E-57 | 53.3 |
| GL, BD and M | -7518.94 | 4 | 15045.89 | 261.952 | 1.31173E-57 | 1.30722E-57 | 53.2 |
| FPT, BD, TCI and M by sex | -7535.16 | 9 | 15088.31 | 304.378 | 8.03817E-67 | 8.01049E-67 | 52.8 |
| FPT, depth and M by sex | -7545.15 | 7 | 15104.30 | 320.366 | 2.71273E-70 | 2.70339E-70 | 52.5 |
| GL, BD and TCI by sex | -7557.85 | 7 | 15129.69 | 345.756 | 8.31838E-76 | 8.28974E-76 | 52.1 |
| GL and BD by sex | -7564.26 | 5 | 15138.53 | 354.59 | 1.00406E-77 | 1.0006E-77 | 51.9 |
| GL, FPT,BD and TCI | -7572.26 | 5 | 15154.51 | 370.578 | 3.38852E-81 | 3.37685E-81 | 51.7 |
| GL, FPT and BD | -7574.46 | 4 | 15156.91 | 372.978 | 1.0206E-81 | 1.01709E-81 | 51.6 |
| BD, TCI and M by sex | -7594.96 | 7 | 15203.91 | 419.978 | 6.35237E-92 | 6.3305E-92 | 51 |
| BD and M by sex | -7606.22 | 5 | 15222.44 | 438.502 | 6.03254E-96 | 6.01178E-96 | 50.7 |
| GL, BD and TCI | -7610.95 | 4 | 15229.90 | 445.962 | 1.44738E-97 | 1.4424E-97 | 50.5 |
| GL and BD | -7613.92 | 3 | 15233.84 | 449.904 | 2.01645E-98 | 2.00951E-98 | 50.5 |
| FPT, BD, TCI and M | -7618.62 | 5 | 15247.23 | 463.298 | 2.4895E-101 | 2.481E-101 | 50.3 |
| FPT, BD and M | -7626.10 | 4 | 15260.19 | 476.258 | 3.8185E-104 | 3.8053E-104 | 50.1 |
| BD, TCI and M | -7667.66 | 4 | 15343.32 | 559.384 | 3.3987E-122 | 3.387E-122 | 48.8 |
| BD and M | -7675.26 | 3 | 15356.52 | 572.586 | 4.6188E-125 | 4.6029E-125 | 48.6 |
| FPT, BD, and TCI by sex | -7746.70 | 7 | 15507.39 | 723.456 | 8.008E-158 | 7.9805E-158 | 46.3 |
| FPT, and BD by sex | -7765.42 | 5 | 15540.84 | 756.906 | 4.3647E-165 | 4.3496E-165 | 45.6 |
| FPT, BD, and TCI | -7788.73 | 4 | 15585.45 | 801.516 | 8.9745E-175 | 8.9436E-175 | 44.9 |
| FPT and BD | -7795.48 | 3 | 15596.95 | 813.016 | 2.8564E-177 | 2.8466E-177 | 44.6 |
| BD and TCI by sex | -7819.70 | 5 | 15649.39 | 865.456 | 1.1712E-188 | 1.1671E-188 | 43.8 |
| GL ,FPT, TCI and M by sex | -7822.89 | 9 | 15663.79 | 879.85 | 8.77E-192 | 8.7398E-192 | 43.7 |
| GL ,FPT and M by sex | -7832.73 | 7 | 15679.47 | 895.532 | 3.449E-195 | 3.4372E-195 | 43.4 |
| BD by sex | -7841.80 | 3 | 15689.61 | 905.672 | 2.1668E-197 | 2.1594E-197 | 43 |
| BD and TCI | -7854.29 | 3 | 15714.58 | 930.644 | 8.1889E-203 | 8.1607E-203 | 42.6 |
| BD | -7860.79 | 2 | 15725.59 | 941.652 | 3.3332E-205 | 3.3218E-205 | 42.4 |
| GL , TCI and M by sex | -7912.44 | 7 | 15838.88 | 1054.946 | 8.3442E-230 | 8.3155E-230 | 40.5 |
| GL and M by sex | -7922.66 | 5 | 15855.31 | 1071.374 | 2.2599E-233 | 2.2521E-233 | 40.2 |
| GL ,FPT and TCI by sex | -7931.19 | 7 | 15876.38 | 1092.442 | 6.015E-238 | 5.9943E-238 | 39.8 |
| GL and FPT by sex | -7943.01 | 5 | 15896.02 | 1112.08 | 3.2726E-242 | 3.2614E-242 | 39.4 |
| GL ,FPT, TCI and M | -7953.31 | 5 | 15916.62 | 1132.688 | 1.0963E-246 | 1.0925E-246 | 39 |
| GL ,FPT and M | -7972.24 | 4 | 15952.48 | 1168.546 | 1.7925E-254 | 1.7863E-254 | 38.3 |
| FPT and M by sex | -7978.57 | 5 | 15967.13 | 1183.196 | 1.181E-257 | 1.1769E-257 | 38.1 |
| GL ,FPT and TCI | -8018.94 | 4 | 16045.89 | 1261.952 | 9.3456E-275 | 9.3134E-275 | 36.5 |
| GL and TCI by sex | -8024.65 | 5 | 16059.31 | 1275.372 | 1.1389E-277 | 1.135E-277 | 36.3 |
| GL by sex | -8036.59 | 3 | 16079.17 | 1295.234 | 5.54E-282 | 5.5209E-282 | 35.8 |
| GL and FPT | -8038.12 | 3 | 16082.25 | 1298.312 | 1.1889E-282 | 1.1848E-282 | 35.8 |
| FPT and M | -8039.42 | 3 | 16084.83 | 1300.894 | 3.2693E-283 | 3.2581E-283 | 35.7 |
| GL , TCI and M | -8040.74 | 4 | 16089.48 | 1305.548 | 3.1905E-284 | 3.1795E-284 | 35.7 |
| GL and M | -8060.80 | 3 | 16127.60 | 1343.666 | 1.6851E-292 | 1.6793E-292 | 34.9 |
| TCI and M by sex | -8071.21 | 5 | 16152.42 | 1368.488 | 6.8645E-298 | 6.8408E-298 | 34.5 |
| GL and TCI | -8115.82 | 3 | 16237.64 | 1453.704 | 0 | 0 | 32.6 |
| TCI and M | -8127.95 | 3 | 16261.90 | 1477.968 | 0 | 0 | 32.1 |
| FPT and TCI by sex | -8131.65 | 5 | 16273.30 | 1489.368 | 0 | 0 | 32 |
| GL | -8136.48 | 2 | 16276.96 | 1493.026 | 0 | 0 | 31.8 |
| FPT and TCI | -8159.14 | 3 | 16324.28 | 1540.346 | 0 | 0 | 30.8 |
| FPT by sex | -8164.17 | 3 | 16334.35 | 1550.41 | 0 | 0 | 30.6 |
| FPT | -8176.88 | 2 | 16357.75 | 1573.816 | 0 | 0 | 30.1 |
| TCI by sex | -8256.20 | 3 | 16518.39 | 1734.458 | 0 | 0 | 26.6 |
| TCI | -8274.49 | 2 | 16552.98 | 1769.044 | 0 | 0 | 25.8 |
